# A retrospective study of multivariable analysis of predictive values of lactate-related ratios on 28-day mortality in intensive care units

**DOI:** 10.1097/MD.0000000000044605

**Published:** 2025-09-26

**Authors:** Veysel Dinç, Döndü Genç Moralar, Oğuz Özakin, Serpil Şehirlioğlu

**Affiliations:** a Anesthesiology and Reanimation, Gaziosmanpaşa Training and Research Hospital, Istanbul, Türkiye.

**Keywords:** 28-day mortality, intensive care, lactate albumin ratio, lactate bicarbonate ratio

## Abstract

In this retrospective research, it was aimed to evaluate predictive values of lactate-related ratios on mortality in intensive care units (ICUs) at multivariable level. A total of 4985 patients who were hospitalized in the Anesthesia Intensive Care Unit of Istanbul Gaziosmanpaşa SUAM between January 2015 and January 2025 were included in the study. Patients’ age, gender, body mass index, hospitalization diagnoses and chronic diseases, lactate, albumin, hemoglobin, potential of hydrogen (pH), HCO₃⁻, base excess, platelets, APACHE II score, and 28-day mortality values were obtained. Lactate albumin ratio (LAR), lactate hemoglobin ratio, lactate pH ratio, and lactate bicarbonate ratio of the patients were analyzed. Age, pH, and LAR parameter means were significantly higher in the non-survival group, whereas blood extracellular fluid mean was significantly higher in the survival group (*P* < .05). Acute renal failure and ketoacidosis were more common in the non-survival group (*P* < .05). Twenty-eight-day mortality was significantly correlated with age (*R* = 0.077; *P* < .01), acute renal failure (*R* = 0.030; *P* < .05), ketoacidosis (*R* = 0.028; *P* < .05), albumin (*r* = −0.117; *P* < .01), base excess in the extracellular fluid (*R* = 0.032; *P* < .05), pH (*R* = 0.036; *P* < .05), LAR (0.057; *P* < .01), and hospitalization duration (*R* = 0.044; *P* < .01). Effects of age (*B* = 0.020; *P* < .01), acute renal failure (*B* = −0.648; *P* < .05), base excess in the extracellular fluid (*B* = 0.024; *P* < .05), pH (*B* = 1.753; *P* < .05), and LAR (*B* = 4.893; *P* < .01) were statistically significant. Although the LAR parameter has a predictive value on 28-day mortality in ICU patients, this is not the case in acute renal failure patients. The LAR variable in acute renal failure patients may have different mechanism than in other ICU patients, and this mechanism may be misleading for LAR. Therefore, further predictive parameters are needed for ICU 28-day mortality in acute renal failure patients.

## 1. Introduction

Mortality in intensive care units (ICUs) is a significant health system problem and also has an important place in clinical patient follow-up.^[1,2]^ The causes of mortality in ICU units vary widely. Therefore, many different variables have been studied to reduce ICU mortality levels. These include factors such as physician management,^[3]^ environmental factors,^[4]^ and the period during which patients are admitted to the ICU,^[5,6]^ in addition to the patient’s condition. The main purpose of all these studies is to estimate ICU mortality levels. Although some of the estimation models are methods such as artificial intelligence,^[7]^ machine learning,^[8]^ clinical studies focus on laboratory, and demographic variables of patients. Among these, lactate is one of the important indicators used in the prediction of mortality and morbidity today.^[9,10]^

Lactate is an indicator of hypoperfusion and anaerobic metabolism.^[11]^ Therefore, systematic studies on lactate have reported that serum lactate level has a significant diagnostic value in hospital deaths.^[12]^ Serum lactate level has been reported as a significant marker in predicting mortality in different patient groups such as infection,^[13]^ sepsis,^[14]^ and trauma.^[15]^ The common result of these studies is that the ratios between lactate and various serum blood values can be used as mortality markers according to different reasons for hospitalization.

Although studies have been conducted on lactate albumin ratio (LAR) in ICUs,^[16–18]^ there are not enough studies analyzing lactate related ratios levels on 28-day mortality multivariately with high patient numbers. The low number of patients in the studies, the focus on a single reason for hospitalization, and the lack of sufficient space for multivariate analyses indicate the gap in the literature on this subject. In order to both respond to this gap and to make recommendations for clinical practice, this study aimed to evaluate predictive values of lactate related ratios on mortality in ICUs at multivariable level.

Studies on LAR in the literature generally reveal that lactate-albumin values are associated with mortality.^[13–18]^ However, no adequate and comprehensive studies were found on 28-day mortality in ICU patients. Based on this, the primary hypothesis of the study was established as follows:

*H*: The LAR parameter may have predictive value in predicting 28-day mortality in ICU patients.

## 2. Methods

### 2.1. Research model

The research was evaluated as a retrospective cohort study. In this context, patient files that met the research criteria of all patients admitted to our hospital’s ICU between January 2015 and January 2025 were retrospectively examined. The research was patterned in descriptive screening and relational screening models.

### 2.2. Patients

The files of patients who were hospitalized in the Anesthesia Intensive Care Unit of Gaziosmanpaşa SUAM, Istanbul between January 2015 and January 2025 were included in the study. The demographic data, diagnoses, chronic disease information, and ICU stay of the patients will be examined. The data to be used for analysis were selected from laboratory results measured within the first 6 hours after admission to the ICU. The inclusion criteria were as follows:

− Adult patients aged 18 years and over.
− Patients who are hospitalized in the ICU and have at least one lactate and albumin measurement within the first 6 hours of admission.
− Patients having necessary research data in their files.
Exclusion criteria was:
− Patients with any condition that could affect the study results.

A minimum requirement of 88 participants was calculated in the analysis conducted using the G*Power 3.1 (Heinrich-Heine-Universität Düsseldorf, Düsseldorf, Germany) program, based on an effect size of 0.30, α = 0.05 and an 80% power level. In the research, 4985 patients were included in the analysis.

**Figure 1.**
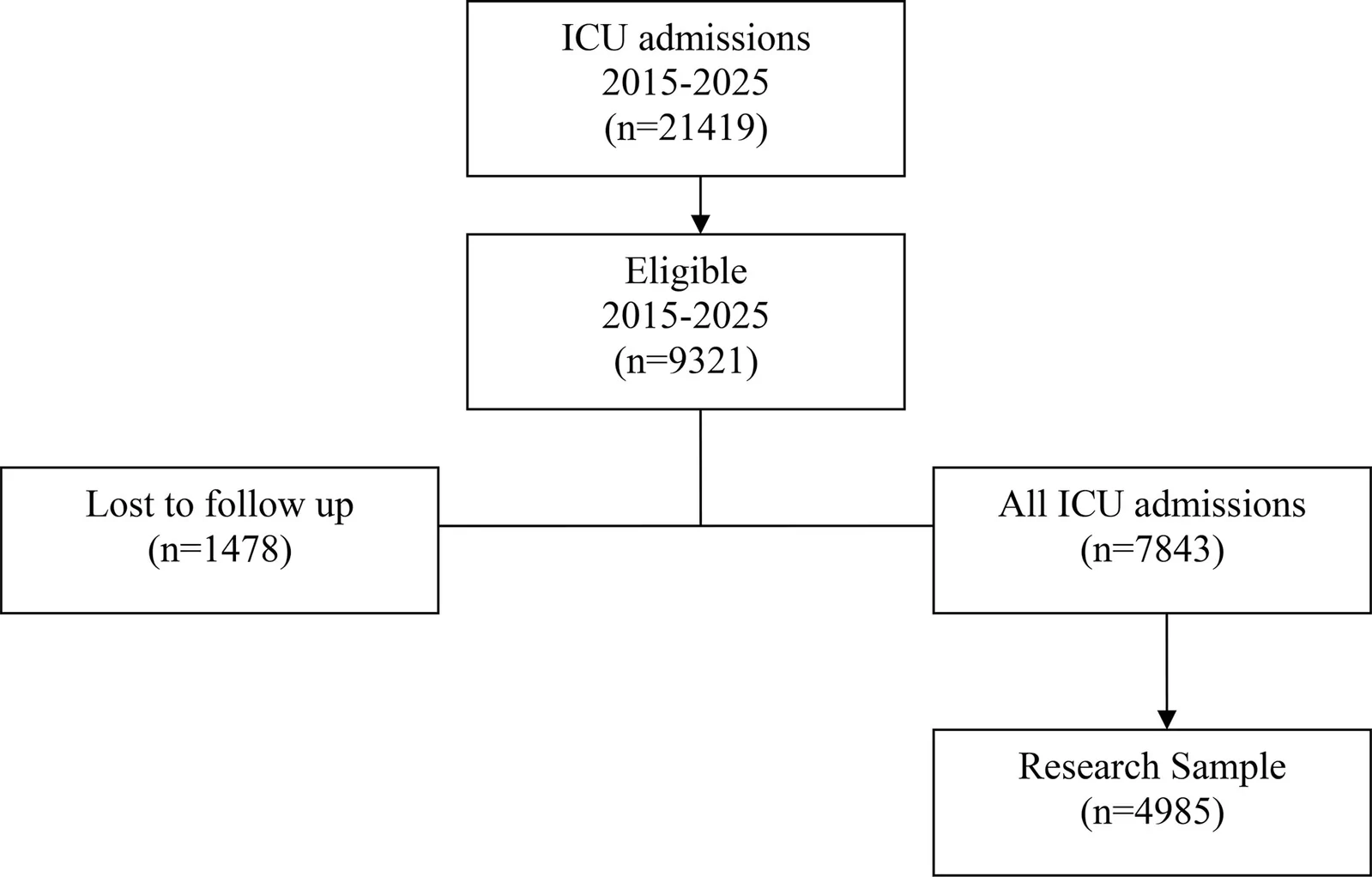
Research flow chart.

A research flow chart was given in Figure 1.

### 2.3. Research data

Patients’ age, gender, body mass index (BMI), hospitalization diagnoses and chronic diseases, lactate, albumin, hemoglobin, potential of hydrogen (pH), HCO₃⁻, base excess, platelets, APACHE II score and 28-day mortality values were obtained. The highest value for lactate in the first 6 hours of ICU, the lowest value for albumin and the values closest in time to the lactate measurement for other variables (hemoglobin, pH, HCO₃⁻) were obtained. The first and 24th hour lactate values and lactate clearance [(initial lactate − 24th hour lactate)/initial lactate] × 100 were calculated.

Research data were collected through the hospital information system without collecting patient personal information, as stipulated in the relevant ethics committee approval and the Declaration of Helsinki guidelines. Patient protocol numbers were used for de-identification purposes. Due to ethics committee approval restrictions, data were not uploaded to an anonymous database.

Since the research was conducted on patient files, patients having missing values in their patient files were excluded from the research and analysis.

### 2.4. Statistical methods

In the study, nominal and ordinal data were defined by frequency analysis. Mean, standard deviation, median, and change intervals were used to define the measurement data. Kolmogorov–Smirnov test was performed for normality distribution. Although nonparametric tests were performed for parameters that did not conform to normal distribution, mean, standard deviation, median, and variation ranges were used in their definitions for a better understanding of the scale parameters. Mann–Whitney *U* test was used in the difference analysis of measurement data, and Fisher Exact test was used in the difference analysis of nominal and ordinal data. In relational screening analysis, and Binary Logistic Regression analysis was used in regression analysis due to linearization deviations.^[19,20]^ Received operational curve analysis was performed for predictive values of ratios. All analyses were performed using SPSS 25.0 (IBM, New york) for Windows program and at a 95% confidence interval and 0.05 significance level.

### 2.5. Ethical approval

Permission was obtained from the Istanbul Medipol University Non-Interventional Clinical Research Ethics Committee for the study. Since the study was retrospective and patient information was not provided, patient consent forms were not obtained.

## 3. Results

The mean age of the survival group was 64.85 ± 18.47, and the mean age of the non-survival group was 70.94 ± 15.42. 46.4% of the survival group and 46.3% of the non-survival group were female. The mean BMI of the survival group was 26.86 ± 6.37, and the mean BMI of the non-survival group was 26.20 ± 5.27.

Age, pH, and LAR parameter means were significantly higher in the non-survival group, whereas blood extracellular fluid mean was significantly higher in the survival group (*P* < .05). Acute renal failure and ketoacidosis were more common in the non-survival group (*P* < .05). Gender, BMI, other hospitalization reasons, comorbidity, and other laboratory parameter differences between patient groups were statistically insignificant (*P* > .05) (Table 1).

**Table 1:** Baseline characteristics of patient groups and difference analysis results.

	Survival (n = 4685; 94.0%)	Non-survival (n = 300; 6.0%)	*P* value	Min effect size	Max effect size
Age, yr, mean ± SD	64.85 ± 18.47	**70.94 ± 15.42**	.000*	0.000	0.000
Median (min–max)	68.00 (19.00–122.00)	**73.00 (22.00–100.00**)			
Gender, n (%)					
Female	2175 (46.4)	139 (46.3)	.512†		
Male	2510 (53.6)	161 (53.7)			
BMI, kg/m^2^, mean ± SD	26.86 ± 6.37	26.20 ± 5.27	.431*		
Median (min–max)	25.31 (10.00–70.76)	25.69 (17.31–62.43)			
Hospitalization reason, n (%)					
Sepsis	315 (6.7)	21 (7.0)	.462†		
Respiratory failure	335 (7.2)	22 (7.3)	.487†		
Trauma	558 (11.9)	34 (11.3)	.426†		
Infection	526 (11.2)	38 (12.7)	.248†		
COPD exacerbation	402 (8.6)	20 (6.7)	.147†		
Brain hemorrage	110 (2.3)	5 (1.7)	.300†		
Malignancy	491 (10.5)	27 (9.0)	.240†		
Acute renal failure	170 (3.6)	**18 (6.0**)	.033†		
Status epileptikus	29 (0.6)	2 (0.7)	.565†		
Ketoacidosis	92 (2.0)	**11 (3.7**)	.044†		
CVE	400 (8.5)	26 (8.7)	.501†		
Inoxication	59 (1.3)	3 (1.0)	.482†		
Acute abdomen	95 (2.0)	9 (3.0)	.172†		
Epilepsy	65 (1.4)	3 (1.0)	.408†		
Cardiac arrest	148 (3.2)	12 (4.0)	.255†		
Heart failure	128 (2.7)	10 (3.3)	.318†		
Other	762 (16.3)	39 (13.0)	.076†		
Comorbidity, n (%)					
HT	2203 (47.0)	131 (43.7)	.142†		
COPD	1104 (23.6)	59 (19.7)	.068†		
Renal failure	792 (16.9)	61 (20.3)	.076†		
Liver failure	38 (0.8)	1 (0.3)	.310†		
Heart failure	585 (12.5)	38 (12.7)	.492†		
Malignancy	721 (15.4)	46 (15.3)	.529†		
Asthma	389 (8.3)	25 (8.3)	.525†		
DM	1457 (31.1)	93 (31.0)	.514†		
Epilepsy	149 (3.2)	7 (2.3)	.268†		
CVE	184 (3.9)	17 (5.7)	.095†		
Laboratory					
Albumin, g/dL, mean ± SD	33.04 ± 7.18	29.31 ± 7.45	.000*	0.000	0.000
Median (min-max)	33.00 (10.00–54.00)	29.00 (7.00–48.00)			
Lactate 6 h, mg/dL, mean ± SD	2.26 ± 1.68	2.35 ± 1.71	.460*	0.221	0.223
Median (min–max)	1.76 (0.06–17.61)	1.82 (0.30–13.21)			
Lactate 24 h, mg/dL, mean ± SD	2.05 ± 1.66	2.15 ± 1.83	.711*	0.337	0.362
Median (min–max)	1.58 (0.00–19.19)	1.59 (0.59–14.11)			
BEecf, mEq/L, mean ± SD	−**1.89 ± 7.37**	−0.88 ± 7.53	.025*	0.010	0.016
Median (min–max)	−**1.60** (−**34.00 to 30.20**)	−0.90 (−28.20 to 30.20)			
Glucose, mg/dL, mean ± SD	157.62 ± 73.77	156.68 ± 77.85	.812*	0.388	0.414
Median (min-max)	140.00 (11.00–845.00)	140.00 (20.00–659.00)			
CHCO3, mg/L, mean ± SD	23.50 ± 6.62	24.21 ± 6.84	.095*	0.041	0.052
Median (min–max)	23.40 (1.50–53.70)	23.90 (4.90–52.90)			
pH	7.36 ± 0.11	**7.38 ± 0.11**	.011*	0.003	0.007
	7.38 (6.63–7.67)	**7.39 (6.89–7.65**)			
PLT, G/L, mean ± SD	238.55 ± 107.65	235.85 ± 130.58	.221*	0.106	0.123
Median (min–max)	224.00 (1.00–1119.00)	219.50 (11.00–1586.00)			
Hb, g/dL, mean ± SD	11.74 ± 2.65	11.64 ± 2.52	.514*	0.248	0.271
Median (min–max)	11.70 (2.20–23.60)	11.50 (5.10–22.50)			
APACHE	38.32 ± 28.72	42.34 ± 31.87	.121*	0.054	0.067
	32.20 (0.00–99.00)	39.00 (0.00–99.00)			
Lactate clearance, %	2.89 ± 45.44	3.35 ± 42.41	.396*	0.181	0.201
	7.94 (−830.00 to 100.00)	9.96 (−243.06 to 83.90)			
LAR	0.07 ± 0.06	**0.09 ± 0.08**	.000*	0.000	0.000
	0.06 (0.00–0.70)	**0.06 (0.01–0.72**)			
LHBR	0.20 ± 0.18	0.22 ± 0.19	.407*	0.192	0.213
	0.15 (0.01–3.06)	0.16 (0.03–1.25)			
LPHR	0.31 ± 0.23	0.32 ± 0.24	.512*	0.245	0.268
	0.24 (0.01–2.55)	0.24 (0.04–1.92)			
LBR	0.12 ± 0.21	0.12 ± 0.19	.983*	0.489	0.515
	0.07 (0.00–6.44)	0.07 (0.01–2.49)			
Hospitalization duration	**7.98 ± 12.45**	7.50 ± 6.91	.002*	0.000	0.002
	**4.00 (1.00–189.00**)	5.00 (1.00–29.00)			

Bold values indicate higher levels.

BEecf = base excess in the extracellular fluid, BMI = body mass index, CHCO3 = bicarbonate, COPD = chronic obstructive pulmonary disease, CVE = cerebrovascular event, DM = diabetes mellitus, HB = hemoglobin, HT = hypertension, LAR = lactate albumin ratio, LBR = lactate bicarbonate ratio, LHBR = lactate hemoglobin ratio, LPHR = lactate pH ratio, pH = potential hydrogene, PLT = platelet, SD = standard deviation.

* Mann–Whitney *U* test.

† Fisher Exact test.

In order to select variables for regression analysis, Spearman rho correlation was performed for both prognostic variables and demographic variables with 28-day mortality.^[19,20]^ Significantly correlated factors with 28-day mortality were selected as regression parameters as univariate screening (Table 2). Binary logistic analysis results showed that effects of age (*B* = 0.020; *P* < .01), acute renal failure (*B* = −0.648; *P* < .05), base excess in the extracellular fluid (BEecf) (*B* = 0.024; *P* < .05), pH (*B* = 1.753; *P* < .05), and LAR (*B* = 4.893; *P* < .01) were statistically significant (Table 3).

**Table 2:** Spearman rho correlation analysis results between 28-day mortality and patient characteristics.

Mortality	*r*	*P*
*Age*	**0.077** **	**.000**
Gender	0.000	.975
BMI	−0.021	.431
Sepsis	0.003	.853
Respiratory failure	0.002	.905
Trauma	−0.004	.765
Infection	0.011	.446
COPD exacerbation	−0.016	.248
Brain hemorrage	−0.011	.446
Malignancy	−0.012	.415
*Acute renal failure*	**0.030** *	**.037**
Status epileptikus	0.001	.919
*Ketoacidosis*	**0.028** *	**.044**
CVE	0.001	.938
Intoxication	−0.006	.694
Acute abdomen	0.016	.253
Epilepsy	−0.008	.575
Cardiac arrest	0.011	.423
Heart failure	0.009	.538
Other	−0.021	.136
HT	−0.016	.259
COPD	−0.022	.122
Renal failure	0.022	.126
Liver failure	−0.013	.363
Heart failure	0.001	.927
Malignancy	0.000	.979
Asthma	0.000	.985
DM	−0.001	.971
Epilepsy	−0.012	.414
CVE	0.021	.138
*Albumin*	−0.117**	**.000**
Lactate 6 h	0.010	.460
Lactate 24 h	0.005	.711
*BEecf*	**0.032** *	**.025**
Glucose	−0.003	.812
CHCO3	0.024	.095
*pH*	**0.036** *	**.011**
PLT	−0.017	.221
HB	−0.009	.514
APACHE	0.022	.121
Lactate clearance	0.012	.396
*LAR*	**0.057** **	**.000**
LHBR	0.012	.407
LPHR	0.009	.512
LBR	0.000	.983
*Hospitalization duration*	**0.044** **	**.002**

Bold values indicate higher levels.

BEecf = base excess in the extracellular fluid, BMI = body mass index, COPD = chronic obstructive pulmonary disease, CHCO3 = bicarbonate, CVE = cerebrovascular event, DM = diabetes mellitus, HB = hemoglobin, HT = hypertension, pH = potential hydrogene, PLT = platelet, LAR = lactate albumin ratio, LBR: lactate bicarbonate ratio, LHBR = lactate hemoglobin ratio, LPHR = lactate pH ratio.

* *P* < .05.

** *P* < .01.

**Table 3:** Binary logistic analysis results for effects of significantly correlated parameters on 28-day mortality.

	*B*	SE	Wald	df	*P*	OR	95% CI for OR	
							Lower	Upper
*Age*	**0.020**	**0.004**	**29.584**	**1**	**.000**	**1.021**	**1.013**	**1.028**
*Acute renal failure*	−**0.648**	**0.260**	**6.236**	**1**	**.013**	**0.523**	**0.314**	**0.870**
Ketoacidosis	−0.604	0.334	3.264	1	.071	0.547	0.284	1.053
*BEecf*	**0.024**	**0.010**	**5.665**	**1**	**.017**	**1.024**	**1.004**	**1.044**
*pH*	**1.753**	**0.692**	**6.424**	**1**	**.011**	**5.772**	**1.488**	**22.389**
*LAR*	**4.893**	**0.831**	**34.659**	**1**	**.000**	**133.295**	**26.147**	**679.526**
Hospitalization duration	−0.007	0.006	1.387	1	.239	0.993	0.982	1.005
Constant	−16.155	5.155	9.819	1	.002	0.000		

Bold values indicate higher levels.

*B* = regression coefficient, BEecf = base excess in the extracellular fluid, CI = confidence interval, df = degree of freedom, LAR = lactate albumin ratio, OR = odds ratio, pH = potential hydrogen, SE = standard error.

**Figure 2.**
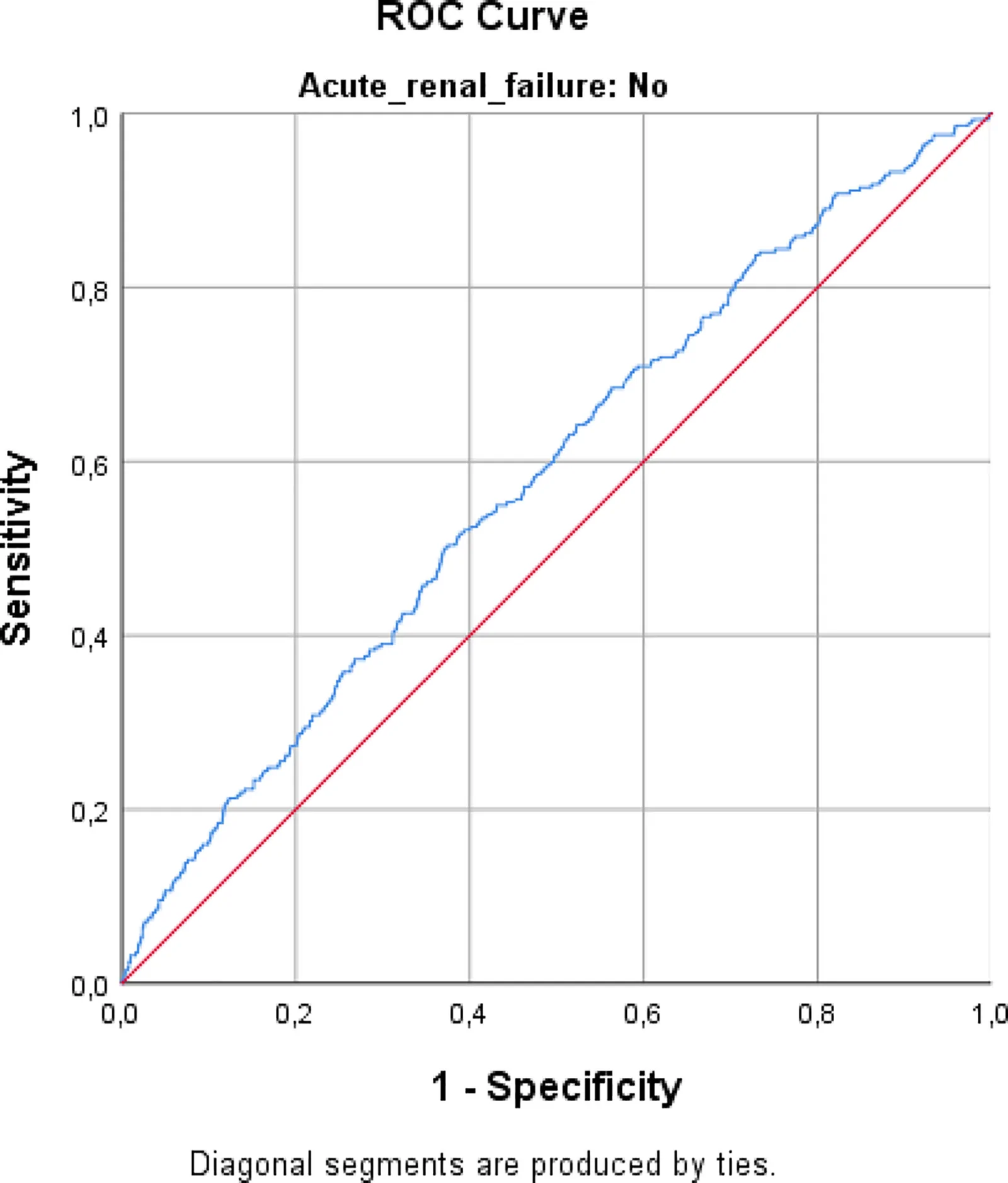
ROC curve for predictive value of LAR on 28-day mortality. LAR = lactate albumin ratio, ROC = received operational curve.

Area under curve (AUC) was 0.569 showing that LAR had 56.9% predictive value on 28-day mortality at ICU (*P* < .05). For lactate level at 0.0554 cut off value, sensitivity was 58.7%, and specificity was 50.1%. For lactate level at 0.0643 cut off value, sensitivity was 49.7%, and specificity was 60.5% (Fig. 2). Received operational curve analysis results also showed that in acute renal failure patients, predictive value for LAR was insignificant (AUC: 0.452; *P* > .05), whereas it was significant for no acute renal failure patients (AUC: 0.581; *P* < .01) with poor predictive ability.

**Figure 3.**
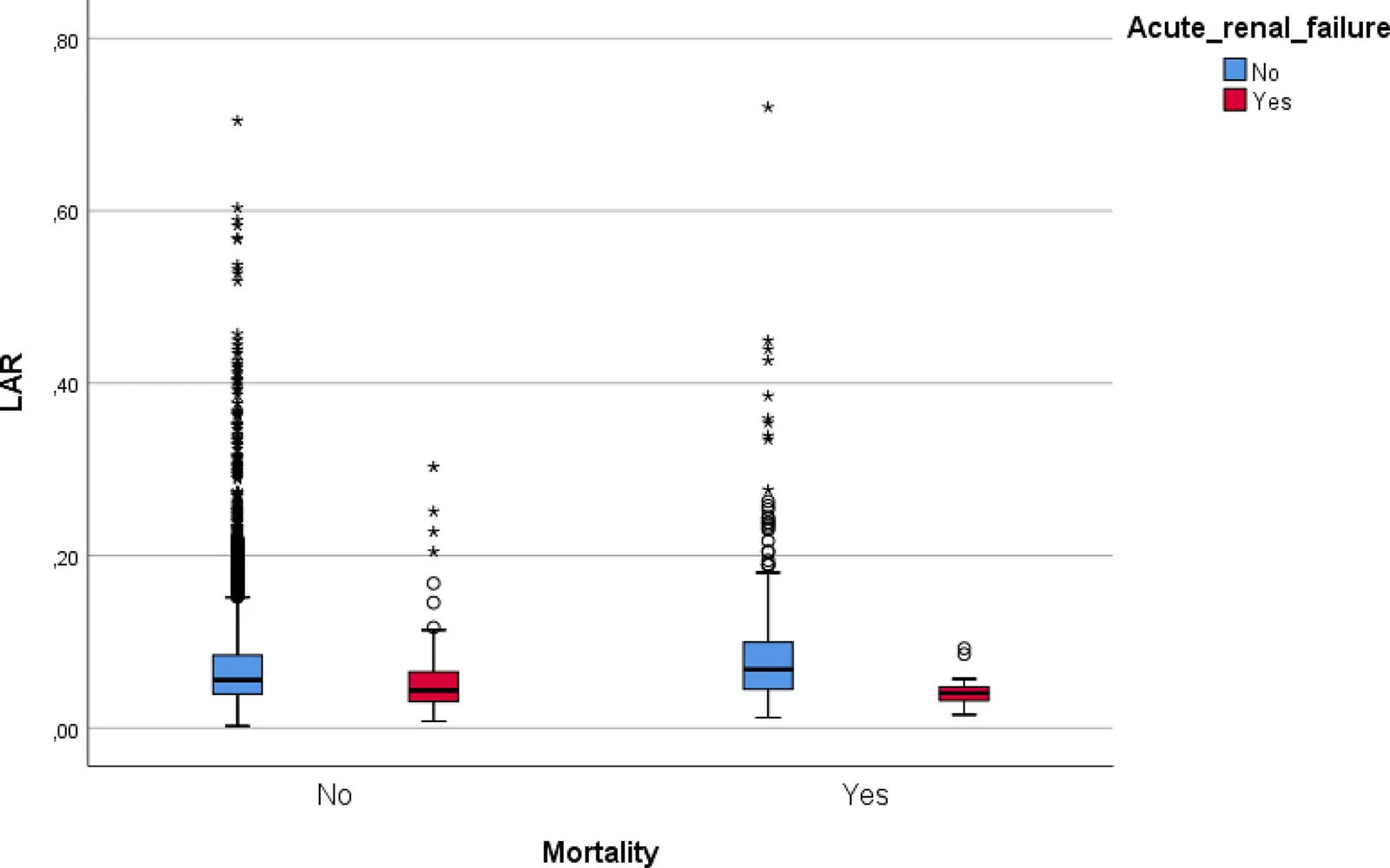
LAR distributions and differences between 28-day mortality groups according to acute renal failure. LAR = lactate albumin ratio.

In patients not having acute renal failure, LAR mean was higher in the 28-day mortality group. In acute renal patients, LAR mean was higher in the survival group (Fig. 3).

## 4. Discussion

In this study, predictive values of lactate related ratios on ICU 28-day mortality levels were analyzed multivariately and among LAR, lactate hemoglobin ratio, lactate pH ratio, and lactate bicarbonate ratio examined in this context, only LAR variable was found to have a significant predictive value on 28-day ICU mortality. In acute renal failure patients, the predictive value of LAR variable were found to be exactly the opposite compared to other age-related causes.

Since ICU mortality rates are high in societies and are an important health problem, studies are being conducted on this subject. There are studies in the literature reporting that ICU mortality rates increase with advanced age.^[21,22]^ Ismail et al^[21]^ and Rodrigues et al^[22]^ reported that mortality rates also increase at advanced ages. In our study, age, pH, LAR parameter means were significantly higher in the non-survival group, whereas blood extracellular fluid mean was significantly higher in the survival group (*P* < .05). Acute renal failure and ketoacidosis were more common in the non-survival group (*P* < .05). The data we obtained in our study were consistent with the literature in this respect. It is possible to state that both the decrease in health parameters and the increase in comorbid diseases at advanced ages are effective on this result. For more advanced results, age-controlled studies may be conducted.

Studies have been conducted on the predictive value of the lactate-albumin ratio on ICU mortality levels, and in general, in these studies, LAR was found to have a significant predictive value for ICU mortality. Islam et al^[23]^ reported in their study that LAR ratio could be an effective biomarker in myocardial infarction patients. In another study, Liu^[24]^ reported the LAR parameter as an important mortality marker for 28-day mortality in ICU critical heart patients. Hua et al^[25]^ reported in their study that LAR value has a predictive value for mortality in critical condition developing with sepsis in acute kidney injury patients. Zhang et al^[26]^ reported the LAR predictive value for 28-day mortality in ICU patients with acute ischemic stroke. In another study, Jin et al^[27]^ reported that LAR parameter has a predictive value on 28-day mortality for patients with myocardial infarction. Suzuki et al^[16]^ conducted a comprehensive study on the national database, scanned 6 years of files for the predictive value of the LAR parameter, and reported that the predictive value was significant. In our study, 28-day mortality was significantly correlated with age (*R* = 0.077), acute renal failure (*R* = 0.030), ketoacidosis (*R* = 0.028), albumin (*r* = −0.117), BEecf (*R* = 0.032), pH (*R* = 0.036), LAR (0.057), and hospitalization duration (*R* = 0.044). Effects of age (*B* = 0.020), acute renal failure (*B* = −0.648), BEecf (*B* = 0.024), pH (*B* = 1.753), and LAR (*B* = 4.893) were statistically significant. Although we obtained similar results with the literature in our study, the situation was different for patients presenting with acute renal failure complaints and the predictive value of the ratios of lactate with other parameters other than LAR on ICU 28-day mortality was not statistically significant. Therefore, further multivariate, multicenter studies are needed, especially for patients with acute renal failure.

### 4.1. Limitations of the study

The most important limitation of the study is that patient follow-ups in the national data collection system are difficult due to the transition of patients between the public and private sectors. Although more comprehensive and extensive follow-up systems have been developed in this regard, compiling and accessing data, especially regarding post-ICU mortality, is seen as a significant limitation. In addition, due to the advanced average age, new comorbidities should be screened in addition to those known from comorbidities. When most patients arrive at the ICU, there may be comorbidities that have not yet been diagnosed.

Although there are studies examining the effects of LAR on ICU mortality, there are not enough studies in the literature that perform multivariate analysis of other lactate rates and LAR rates according to different reasons for hospitalization. Therefore, the lack of sufficient studies to compare the research results in a multivariate dimension is another limitation of the study.

In general, potential bias from the retrospective design, limited generalizability due to the single-center setting, and the weak predictive performance of LAR may be important limitations of the study.

### 4.2. Contribution of the research to literature and clinical applications

The most important contribution of the study to the literature is that it also examines other indicators related to lactate and, unlike studies on LAR, it performs a multivariate analysis. In this way, the study addressed the predictive effect of LAR on ICU 28-day mortality in a multivariate and multidimensional manner. In this respect, the status of the LAR parameter in patients with acute renal failure among the findings obtained in the study can be considered an important finding for the literature. In addition, the scanning of the entire archive of a state hospital for 10 years also makes a significant contribution to the literature in terms of sample size.

The most important contribution of the study to clinical practice is that it reveals that the LAR variable for acute renal failure has no predictive value on ICU 28-day mortality. In this context, when making an evaluation with LAR in clinical follow-ups, it is necessary to question whether the patient has acute renal failure. If the patient has acute renal failure, then LAR is not a reliable predictive parameter for ICU mortality.

Although studies on the LAR have been conducted in ICUs, there are insufficient studies examining the lactate-related ratio levels on 28-day mortality using multivariate data with large patient populations. In this respect, this study demonstrates novelty both in its largest sample size of 4985 patients and its multivariate approach.

## 5. Conclusion

In ICU patients generally, the LAR parameter has a predictive value on 28-day mortality; however, in patients with acute renal failure, this is not the case. The LAR variable may have a different mechanism in patients with acute renal failure than in other patients in the ICU, and this mechanism may be deceptive for LAR. For ICU 28-day mortality in patients with acute renal failure, additional prognostic indicators are therefore required.

## Acknowledgments

We thank Kadir Yilmaz for his valuable help with statistical analysis.

## Author contributions

**Conceptualization:** Veysel Dinç.

**Formal analysis:** Veysel Dinç.

**Investigation:** Oğuz Özakin, Serpil Şehirlioğlu.

**Methodology:** Veysel Dinç, Döndü Genç Moralar, Oğuz Özakin, Serpil Şehirlioğlu.

**Supervision:** Veysel Dinç, Döndü Genç Moralar.

**Writing – original draft:** Veysel Dinç.

**Writing – review & editing:** Veysel Dinç.

## References

[1] Pronovost PJ Rinke ML Emery K Dennison C Blackledge C Berenholtz SM. Interventions to reduce mortality among patients treated in intensive care units. J Crit Care. 2004;19:158–64. 10.1016/j.jcrc.2004.07.003 15484176

[2] Rocker G Cook D Sjokvist P. Clinician predictions of intensive care unit mortality. Crit Care Med. 2004;32:1149–54. 10.1097/01.ccm.0000126402.51524.52 15190965

[3] Levy MM Rapoport J Lemeshow S Chalfin DB Phillips G Danis M. Association between critical care physician management and patient mortality in the intensive care unit. Ann Intern Med. 2008;148:801–9. 10.7326/0003-4819-148-11-200806030-00002 PMC2925263 18519926

[4] Melaku EE Urgie BM Dessie F Seid A Abebe Z Tefera AS. Determinants of mortality of patients admitted to the intensive care unit at debre berhan comprehensive specialized hospital: a retrospective cohort study. Patient Relat Outcome Meas. 2024;15:61–70. 10.2147/PROM.S450502 PMC10895994 38410832

[5] Finkielman JD Morales IJ Peters SG. Mortality rate and length of stay of patients admitted to the intensive care unit in July. Crit Care Med. 2004;32:1161–5. 10.1097/01.ccm.0000126151.56590.99 15190967

[6] Wunsch H Mapstone J Brady T Hanks R Rowan K. Hospital mortality associated with day and time of admission to intensive care units. Intensive Care Med. 2004;30:895–901. 10.1007/s00134-004-2170-3 15007545

[7] Olang O Mohseni S Shahabinezhad A. Artificial intelligence-based models for prediction of mortality in ICU patients: a scoping review. J Intensive Care Med. 2024;1:08850666241277134. 10.1177/08850666241277134 39150821

[8] Ali G Al-Kafi GA Faiza JT Hoque MI Suha SA. Revolutionizing intensive care: a machine learning based approach for ICU patients’ in-hospital mortality prediction. In: 2024 6th International Conference on Electrical Engineering and Information & Communication Technology (ICEEICT). 2024:711–716. IEEE.

[9] Tomruk M Balasar B Eroğlu E. The effects of blood lactate level on mortality in patients developing sepsis in intensive care unit. Anatolian Clin J Med Sci. 2025;30:78–81.

[10] Duan W Yang F Ling H Li Q Dai X. Association between lactate to hematocrit ratio and 30-day all-cause mortality in patients with sepsis: a retrospective analysis of the Medical Information Mart for Intensive Care IV database. Front Med. 2024;11:1422883. 10.3389/fmed.2024.1422883 PMC11347292 39193015

[11] Casserly B Phillips GS Schorr C. Lactate measurements in sepsis-induced tissue hypoperfusion: results from the Surviving Sepsis Campaign database. Crit Care Med. 2015;43:567–73. 10.1097/CCM.0000000000000742 25479113

[12] Kruse O Grunnet N Barfod C. Blood lactate as a predictor for in-hospital mortality in patients admitted acutely to hospital: a systematic review. Scandinavian J Trauma Resuscitation Emerg Med. 2011;19:1–12. 10.1186/1757-7241-19-74 PMC3292838 22202128

[13] Trzeciak S Dellinger RP Chansky ME. Serum lactate as a predictor of mortality in patients with infection. Intensive Care Med. 2007;33:970–7. 10.1007/s00134-007-0563-9 17431582

[14] Mikkelsen ME Miltiades AN Gaieski DF. Serum lactate is associated with mortality in severe sepsis independent of organ failure and shock. Crit Care Med. 2009;37:1670–7. 10.1097/CCM.0b013e31819fcf68 19325467

[15] Odom SR Howell MD Silva GS. Lactate clearance as a predictor of mortality in trauma patients. J Trauma Acute Care Surg. 2013;74:999–1004. 10.1097/TA.0b013e3182858a3e 23511137

[16] Suzuki Y Aoki Y Shimizu M. Predictive accuracy of lactate albumin ratio for mortality in intensive care units: a nationwide cohort study. BMJ open. 2024;14:e088926. 10.1136/bmjopen-2024-088926 PMC11667448 39806598

[17] Zeng Z Huang R Lin H Peng H Luo J Ding N. Serum lactate is an indicator for short-term and long-term mortality in patients with acute pancreatitis. Dig Dis Sci. 2024;69:2223–34. 10.1007/s10620-024-08419-4 38594436

[18] Chen M Lv D. Prognostic value of serum lactate level for mortality in patients with acute kidney injury. Eur J Med Res. 2024;29:295. 10.1186/s40001-024-01886-5 PMC11110292 38778420

[19] Yilmaz K Turanli M. A multi-disciplinary investigation of linearization deviations in different regression models. Asian J Probability Stat. 2023;22:15–9.

[20] Yilmaz K Turanli M. A multi-disciplinary investigation on minimizing linearization deviations in different regression models. Change & Shaping The Future, IV. ASC-2022/Fall Congress. ISBN 978-625-8048-99-5.

[21] Ismail AJ Hassan WMNW Nor MBM Shukeri WFWM. The impact of age on mortality in the intensive care unit: a retrospective cohort study in Malaysia. Acute Critical Care. 2024;39:390–9. 10.4266/acc.2024.00640 PMC11392691 39266274

[22] Rodrigues AR Oliveira A Vieira T. A prolonged intensive care unit stay defines a worse long-term prognosis – insights from the critically ill mortality by age (Cimba) study. Aust Crit Care. 2024;37:734–9. 10.1016/j.aucc.2024.03.001 38649316

[23] Islam MU Gollapinni U Hassan SU. Lactate-to-albumin ratio (LAR) as a predictor of all-cause mortality in patients with myocardial infarction: a systematic review and meta-analysis. Cureus. 2025;17:1. 10.7759/cureus.82166 PMC12070447 40364881

[24] Liu Y. Association between lactate/albumin ratio and 28-day mortality in ICU critical patients with coronary heart disease: a retrospective analysis of the MIMIC-IV database. Front Cardiovasc Med. 2024;11:1486697. 10.3389/fcvm.2024.1486697 PMC11609210 39624213

[25] Hua Y Ding N Jing H. Association between the lactate-to-albumin ratio (LAR) index and risk of acute kidney injury in critically ill patients with sepsis: analysis of the MIMIC-IV database. Front Physiol. 2025;16:1469866. 10.3389/fphys.2025.1469866 PMC11879934 40046181

[26] Zhang S Chen N Ma L. Lactate-to-albumin ratio: a promising predictor of 28-day all-cause mortality in critically Ill patients with acute ischemic stroke. J Stroke Cerebrovasc Dis. 2024;33:107536. 10.1016/j.jstrokecerebrovasdis.2023.107536 38636322

[27] Jin P Bian Y Cui Q Liang X Sun Y Zheng Q. Association between lactate/albumin ratio and 28-day all-cause mortality in critically ill patients with acute myocardial infarction. Sci Rep. 2024;14:23677. 10.1038/s41598-024-73788-9 PMC11466948 39389996

